# Understanding older adults’ attitudes toward mobile and wearable technologies to support health and cognition

**DOI:** 10.3389/fpsyg.2022.1036092

**Published:** 2022-12-08

**Authors:** Ibukun E. Fowe, Walter R. Boot

**Affiliations:** ^1^Department of Psychology, Florida State University, Tallahassee, FL, United States; ^2^Institute for Successful Longevity, Florida State University, Tallahassee, FL, United States

**Keywords:** technology acceptance, technology adoption, telehealth, remote monitoring, aging, digital divide

## Abstract

The use of technology to facilitate remote patient monitoring and virtual care is desirable due to the challenges of providing healthcare during the COVID-19 pandemic and the need for more efficient and effective methods to care for the expanding older adult population. Further, the collection and sharing of patient generated health data (PGHD) through these technologies holds promise with respect to improving outcomes and reducing the cost of care by facilitating the early detection and treatment of cognitive and health problems. Despite the potential benefits of these technologies, their promise might be hampered by low rates of acceptance and adoption among older adults. In an online survey, we assessed community-dwelling older adults’ (*N* = 92) attitudes towards the use of wearable and mobile technologies for (1) predicting cognitive decline, (2) assisting with adherence to healthy activities, and (3) collecting self-report data to understand current and predict future health states. Participants generally agreed hypothetical technology solutions would be useful (*M* = 4.20, *SD* = 0.70 on a 1–5 agreement scale; 5 = “strongly agree”), that they were interested in learning more about these technologies (*M* = 4.04, *SD* = 0.74), and that they would be willing to adopt these technologies (*M* = 3.83, *SD* = 0.93), though attitudes varied. Although participants were generally positive toward these technologies, they were relatively neutral in terms of their agreement that privacy of generated data was a concern (*M* = 2.92, *SD* = 1.02). Privacy concerns were associated with lower interest and willingness to adopt. More positive general technology attitudes and higher mobile device proficiency were associated with greater acceptance and willingness to adopt these technologies. Finally, poorer self-rated health was related to negative attitudes toward these technologies. These findings highlight barriers and potential targets for intervention to increase uptake of these and similar technologies among older adults who may be reluctant to adopt remote monitoring technologies.

## Introduction

The use of technologies that facilitate remote patient monitoring and care has increased substantially over the past few years in the United States, with analyses suggesting that telehealth usage increased approximately 38 times from usage observed during the early part of the year 2020 (Bestsennyy et al., 2021). The challenges of providing healthcare during the COVID-19 pandemic and the need for more efficient and effective methods to care for the rapidly aging population have accelerated this trend (Hoffman, 2020; Hosseinzadeh et al., 2020; Schulz et al., 2022). Mobile and wearable technologies specifically have the potential to support the early detection of, and intervention for, a variety of age-related health conditions by continuously monitoring health and cognitive status through actively or passively produced patient generated health data (PGHD) (Kim et al., 2021). Actively generated PGHD, including health survey data, patient reported outcome measures (PROMS), and other types of patient initiated sharing of health information can facilitate timely and actionable health decisions (Jayakumar et al., 2020). Passively collected PGHD obtained *via* patients’ interactions with devices (e.g., wearables) generate digital phenotypes and biomarkers that can provide additional insights into disease trends and alert providers about the need to take action to prevent poor outcomes (Jayakumar et al., 2020; Waring and Majumder, 2020; Milne et al., 2022). However, the fulfillment of these promises is contingent upon the willingness of patients to share their personally sourced data with health providers for use in assessing their health and cognitive status to help prevent future problems. Privacy and security concerns related to the sharing of health data are potential barriers for older adults (e.g., Fischer et al., 2014; Young et al., 2014), and as will be expanded upon later, adoption and adherence to such technologies to track and share health data might be strongly influenced by age-related differences in technology adoption and proficiency.

Despite the potential benefits of wearable and mobile devices to support older adults’ health and cognition, older adults may face particular barriers in using and adopting these devices, diminishing the potential of these technologies to support healthy aging (e.g., Preusse et al., 2017; Lazaro et al., 2020; Chandrasekaran et al., 2021). This fact is consistent with a still persistent “digital divide” in the United States and around the world, for example in the United States about 25% of older adults (65+) are not online, and approximately 40% do not own a smartphone, compared to near universal adoption and ownership of these technologies among younger adults (Pew Research Center, 2022). The digital divide became especially salient during the COVID-19 pandemic as many vital services, including healthcare services, were shifted online (Xie et al., 2020). Across the literature, however, there is limited knowledge about older adults’ willingness to collect their health-related data from wearable and mobile devices and share this data with healthcare providers (Farivar et al., 2020; Chandrasekaran et al., 2021). Few empirical studies have assessed the attitudes of older adults toward collecting and using their digital data to measure and predict their health and cognitive status, specifically. To facilitate the collection of these data among the older adult population, it is important to understand older adults’ attitudes. It is also important to understand how individual difference characteristics might affect their perceptions.

Models of technology acceptance such as the Technology Acceptance Model (TAM), the Senior Technology Acceptance Model (STAM), and the Unified Theory of Acceptance and Use of Technology (UTAUT) have endeavored to help elucidate the factors that contribute to technology acceptance, providing clues as to factors to target to facilitate adoption (Davis, 1989; Venkatesh et al., 2003; Chen and Chan, 2014; Charness and Boot, 2016; Mitzner et al., 2019). Two key predictors of technology acceptance across these models, including models developed specifically to explain the adoption of technology among older adults, are *perceived usefulness* and *perceived ease of use* of the technology. According to the original definitions provided by Davis (1989), perceived usefulness is defined as “the degree to which a person believes that using a particular system would enhance his or her job performance.” Perceived ease of use refers to “the degree to which a person believes that using a particular system would be free of effort.” More generally, perceived usefulness refers to the anticipated benefit a technology has to achieve a goal (e.g., maintaining health and cognition), and perceived ease of use refers to whether an individual anticipates use of the technology will be easy compared to difficult. Other identified factors include age, level of education, income level, race/ethnicity, gender, prior experience with technology, and self-efficacy (Davis, 1989; Mitzner et al., 2019). These models, particularly The TAM and its various modifications, emphasize perceived usefulness as key to technology acceptance (Alwahaishi and Snášel, 2013). This study posits that perceived usefulness may also be influenced by the general health and cognitive status of the user, with users who are more vulnerable or who have poorer health and cognition more likely to perceive technologies to be more useful compared to their less vulnerable and healthier counterparts. This may be especially relevant to privacy concerns, as older adults may be willing to sacrifice some level of privacy to support their independence through better overall health and cognition.

The current study examined the willingness of older adults to collect their own digital data actively and passively for assessing their current health status and predicting and preventing future problems such as cognitive decline, as well as their willingness to recommend these technologies to others. The potential impact of vulnerability to health and cognitive problems on judgments was evaluated in two ways – 1) by having some participants read about hypothetical individuals who were presented as more or less susceptible to disease, and 2) by comparing participants with better and worse self-rated health and cognition. Concepts such as perceived usefulness and ease of use are anticipated to be important. It is predicted that the vulnerability of the person described in hypothetical scenarios would impact how useful participants would perceive that technology would be for them (greater vulnerability being associated with greater perceived usefulness), resulting in more positive attitudes (similarly, participants’ own health and cognitive status might influence their perceptions of the usefulness of the technology for themselves). Separately, participants’ own proficiency using technology is anticipated to be important as mastery of technology is anticipated to impact perceived ease of use of technologies described.

### Objectives

Study aims were (1) To assess older adults’ attitude toward the use of digital phenotypic or biomarker data from wearable or mobile devices to generate health related predictions about participants’ daily routine to support adherence to healthy behaviors or to predict participants’ likelihood of developing future health or cognitive problems, (2) To assess older adults’ attitude toward the use of wearable or mobile technologies such as smartwatches or smartphones to collect participants’ health related surveys that can be shared with health care providers, and (3) To determine whether health vulnerability has an effect on older adults’ perceptions of these technologies. We hypothesized that older adults would be more positive toward these technologies if the person described in the scenario was described as more vulnerable to health, cognitive, or adherence challenges.

## Materials and methods

### Participants

Data were collected from older adults who were members of Florida State University’s Institute for Successful Longevity’s participant registry.^1^ This registry contains contact information of over 2,500 older adults (age 60 or older) who expressed interest in being study volunteers by responding to an advertisement campaign that included newspaper advertisements, community outreach, and direct mailings. Registry members live in Tallahassee, Florida, or the surrounding region.

Our goal was to obtain responses from approximately 100 individuals. Although this goal was based primarily on study resources, even with some attrition and missing data, the planned sample size was powered to detect at least a medium-sized association (*r* = 0.30) between individual difference characteristics and self-reported attitudes toward the described technologies (alpha = 0.05, power = 0.80), and could detect a medium-large effect (*f* = 0.325) of vulnerability on attitudes (between participant factor) in a two-group ANOVA (Faul et al., 2007).

An email asking for participation in the survey study was sent to approximately 500 individuals in the registry (in two batches of 250) assuming a response rate of approximately 20%. This list of 500 older adults was generated randomly by the Institute for Successful Longevity. Individuals who completed the survey were entered into a raffle to win one of two $50 gift certificates. Of the 108 older adults who initially started the survey, 92 participants completed all survey sections. Their average age was 71.23 years (*SD* = 4.44), and the sample was 59% female. Due to the volunteer nature of the sample and the demographics of the registry, the sample was largely from a high socioeconomic status background. The sample was mostly white and non-Hispanic (92%), most reported an income of $60,000 or more (80%), and most had a bachelor’s degree or higher (78%).

### Materials

The survey was administered using the Qualtrics survey platform, and contained the following sections in order:

#### Health technology scenarios

Participants were presented with three hypothetical health technology scenarios (See Supplemental materials). Scenario 1 asked participants to consider a smartwatch capable of predicting future cognitive decline. Scenario 2 asked participants to consider a smartwatch capable of supporting healthy behaviors by providing reminders based on machine learning predictions of when the wearer of the watch would be most available to engage in the health behaviors. Scenario 3 asked participants to consider a health survey platform administered *via* smartphone that would allow for the diagnosis of current health problems and prediction of future health problems. Half of all participants received information to suggest the person in the hypothetical scenario was vulnerable or susceptible to a health problem relevant to the technology under discussion (e.g., for the smartwatch to predict cognitive decline: “Cindy is a 65-year-old woman” vs. “Cindy is a 65-year-old woman with a family history of Alzheimer’s disease”).

For each technology scenario, participants were asked whether they agreed with statements that 1) the technology would be useful to the person described in the scenario (Useful), 2) they would recommend the technology to the person described in the scenario (Recommend), 3) the person in the scenario should be concerned about their privacy when using the technology (Privacy Concern), 4) the participant themself was interested in learning more about the technology (Interested), and 5) the participant themselves would consider adopting the described technology (Adopt). A Likert scale was presented with the following options; (1) Strongly Disagree, (2) Disagree, (3) Neutral, (4) Agree, (5) Strongly Agree.

#### Mobile device proficiency questionnaire

The MDPQ was administered to understand whether technology proficiency is associated with attitudes toward wearable and mobile technologies to promote physical and cognitive health. The abbreviated (16 item) version of the MDPQ was administered which asked participants to rate their proficiency using mobile devices, including smartphones and tablet computers (e.g., “Using a mobile device I can: Send emails”). This measure has demonstrated validity and reliability (Roque and Boot, 2018; Moret-Tatay et al., 2019; Petrovcic et al., 2019). The measure consists of eight subscales (subscale scores range from 1 to 5 with lower numbers representing lower proficiency). As the short form was used here, each subscale featured two questions which are averaged to produce a subscale score according to the published scoring scheme. Total MDPQ scores are generated by adding all subscales, producing scores that from 8 (lowest proficiency across all subscales) to 40 (highest proficiency).

#### Technology readiness index

The TRI (Parasuraman and Colby, 2014) was administered to understand whether general attitudes toward technology can predict specific attitudes toward wearable and mobile devices to promote physical and cognitive health. The TRI features 16-items and subscales (4 questions each) include technology optimism, innovativeness, discomfort, and insecurity. Technology optimism and innovativeness are scales that represent positive attitudes toward technology, and technology discomfort and insecurity capture negative attitudes. For the current study, total TRI score (an average of subscale scores after reverse coding negative questions) was used with scores ranging from 1 (negative attitudes) to 5 (positive attitudes).

#### Health literacy

Health literacy was another predictor included to understand attitudes toward these health technologies. A brief health literacy measure (3 items) assessed difficulty learning about medical conditions, interpreting medical information, and filling out medical forms (Chew et al., 2004). Participants were asked about the frequency with which difficulties in these domains occurred, from Never (1) to Always (5). Participants who did not report a 3 or above on any question were classified as having high health literacy.

#### Health status

Five questions from the SF-36 assessed health, including questions about general health, chronic diseases, bodily pain, and physical limitations (Brazier et al., 1992). For analysis purposes, however, the general health question (In general, would you say your health is: poor, fair, good, very good, excellent) was used to represent global health given that this question has been found to be as valid, reliable, and sensitive as multi-item scales (Macias et al., 2015) and has been found to be comparable with longer instruments in terms of predicting important health outcomes such as healthcare utilization, hospitalization, and mortality (DeSalvo et al., 2005).

#### Cognitive health

A five-item Perceived Deficits Questionnaire [PDQ-5; adapted from (Sullivan et al., 1990)] was used to assess participants’ cognitive health. Participants were asked to rate how often in the past 4 weeks they had encountered problems with memory, attention, or concentration from 0 = Never to 4 = Almost always. Answers were summed to produce a score from 0 (low cognitive deficits) to 20 (high cognitive deficits).

#### Demographics.

Finally, a brief demographics survey asked participants about their birth year, gender (male: 1; female: −1), race (White: 1; Black or African American: 2; American Indian or Alaska Native: 3; Asian: 4; Native Hawaiian or Pacific Islander: 5; Other: 6; Prefer not to answer: 7), ethnicity (Spanish/Hispanic/Latino: 1; Non-Spanish/Hispanic/Latino: 2), education (<High School: 1; High School/GED: 2; Some College: 3; Associates or Technical Degree: 4; Bachelor’s: 5; Graduate or Professional Degree: 6; Prefer Not to Say: 7), and income (Less than $10,000: 1; $10,000−$19,999: 2; $20,000−$39,999: 3; $40,000−$59,999: 4; $60,000−$79,999: 5; $80,000 or more: 6; Do not know for certain: 7; Do not wish to answer: 8). Birth year was used to calculate an approximate Age variable. For analysis purposes, a Race/Ethnicity variable was created in which White Non-Hispanic individuals were coded as 0, and all other participants were coded as 1. A High Income variable was coded such that individuals reporting an income of $60 K or greater were coded as 1 (less than $60 K as 0). A High Education variable was coded such that individuals earning a Bachelor’s Degree or higher were coded as 1 (less than Bachelor’s 0). If participants selected responses such as “Prefer not to answer,” “Do not know for certain,” or “Do not wish to answer,” these responses were coded as missing data and excluded from reported analyses.

### Procedures

Data collection occurred during April and May of 2022. Emails were sent to individuals within the ISL participant registry with an explanation of the study and link to the survey instrument. After indicating consent to participate within the survey, participants completed the survey instruments in the above order. Qualtrics alternated survey version such that half of the sample received a version of the technology scenarios with no information about the person described in the scenario other than their name, gender, and age, and half received additional information related to their increased vulnerability to health challenges. After completing the survey, participants were directed to a link to a separate Qualtrics form in which they could provide their contact information to be entered into the gift certificate raffle.

## Results

### Participant attitudes toward technologies

Participants were asked about their attitudes toward (1) a smartwatch to predict future cognitive decline, (2) a smartwatch to support adherence to healthy behaviors, and (3) a smartphone app to collect health information to assist with disease diagnosis and the prediction of future health problems. Although we predicted that participants would be more positive toward these technologies and less concerned about privacy when scenarios featured individuals with greater disease vulnerability or susceptibility, initial analyses provided little evidence for this hypothesis.

**Figure 1:**
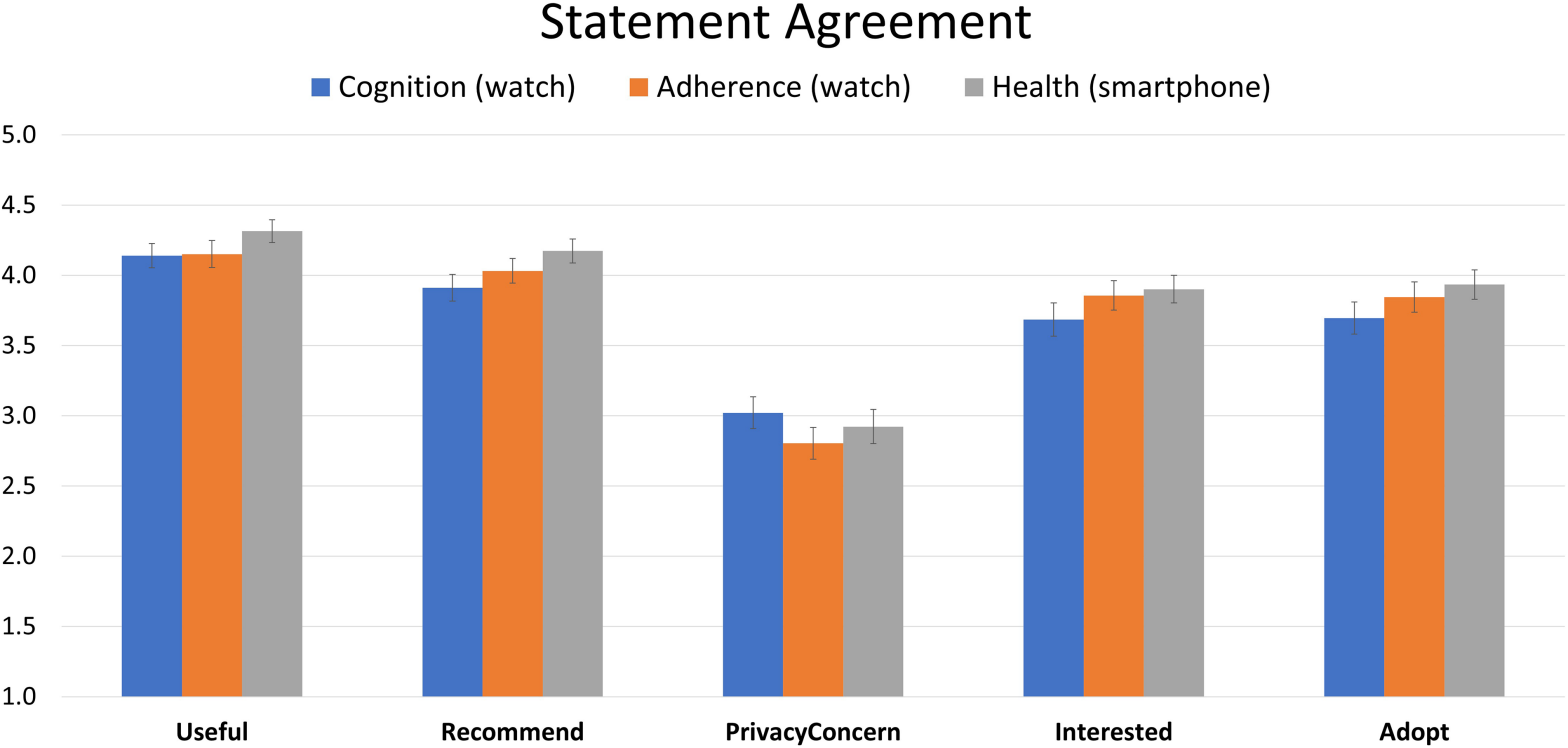
Average participant responses related to three different hypothetical technologies to support cognition, adherence, and overall health. Error bars represent +/- 1 SEM.

Of initial interest was agreement responses related to questions asked of each scenario. These data were entered into an ANOVA with scenario (Cognition, Adherence, Health) and question (Useful, Recommend, Privacy, Interest, Adoption) as within-participant factors, and vulnerability (Not Vulnerable vs. Vulnerable) as a between-participant factor revealed no main effect of vulnerability (*F*(1, 87) = 0.033, *p* = 0.857, *η_p_^2^* = 0.027). Nor did vulnerability interact with scenario, question, or both scenario and question (all *p* values >0.31). This primary planned analysis was supplemented with a MANOVA conducted on participants’ responses to the 15 questions across three scenarios with vulnerability as a between-participant factor. Again, no hint of a vulnerability effect was observed (Wilks’ *Λ* = 0.839, *p* = 0.534). As a result, we collapsed data across vulnerability category. Collapsed data are presented in Figure 1.

#### Usefulness

As can be seen from Figure 1, participants rated all technology solutions as well above neutral (3) in terms of usefulness to the individual described in the scenario. Participants did not rate any technology as significantly more useful than another (*F*(2,182) = 2.499, *p* = 0.085, *η_p_^2^* = 0.009).

#### Recommend

Participants rated all technology solutions as well above neutral (3) in terms of recommending the use of the technology to the individual described in the scenario. Ratings differed between technologies (Figure 1; *F*(2,180) = 5.405, *p* < 0.01; *η_p_^2^* = 0.014). Contrasts found that participants demonstrated a stronger preference to recommend the app to support health (*M* = 4.17, *SD* = 0.82) over the smartwatch to predict cognitive decline (*M* = 3.91, *SD* = 0.90; *p* < 0.01, Cohen’s *d* = 0.34; Bonferroni correction).

#### Privacy concerns

Participants generally neither agreed nor disagreed that individuals described should have privacy concerns when using these three pieces of technology (Figure 1). Responses were close to “neutral” for all technologies, though there were significant differences as a function of technology type (*F*(2,182) = 3.599, *p* < 0.05; *η_p_^2^* = 0.006). This was driven by more privacy concerns for assessing cognition compared (*M* = 3.02, *SD* = 1.09) to supporting adherence (*M* = 2.80, *SD* = 1.08; *p* < 0.01, Cohen’s *d* = 0.38; Bonferroni correction).

#### Interest

Participants were above neutral, on average, in their interest in learning more about these three different technologies to support cognition, adherence, and health. Interest did not differ based on technology type (Figure 1; *F*(2,180) = 2.48, *p* = 0.086; *η_p_^2^* = 0.008).

#### Adoption

Participants were generally positive (above neutral) in terms of considering adoption of the technologies themselves. Attitudes differed significantly between technologies (Figure 1; *F*(2,180) = 4.219, *p* < 0.05; *η_p_^2^* = 0.009). Participants were more positive with respect to adoption for the technology to support health (*M* = 3.93, *SD* = 1.00) over cognition (*M* = 3.70, *SD* = 1.10; *p* < 0.05, Cohen’s *d* = 0.24; Bonferroni correction).

### Associations with attitudes toward technologies to support health and cognition

Next, we examined factors that correlate with participants’ attitudes toward these technologies. To simplify analyses, all questions regarding hypothetical technology scenarios were entered into a Principal Components Analysis (PCA). A two-factor solution (using Varimax Rotation) was found, explaining 67% of observed variance. All privacy concerns loaded onto one factor. Attitudes regarding technology usefulness, interest, whether participants would recommend the technology, and whether they would consider adopting the technology themselves loaded onto another factor. Here, we label these factors as Privacy Concerns and Positive Attitudes with respect to the use of mobile and wearable technologies to support health and cognition.

#### Relations among variables

Table 1 represents Pearson correlations among main variables of interest. A few significant relationships are of note. First, no variables appeared associated with privacy concerns related to the described technologies. However, more positive general attitudes toward technology (as measured by the Tech Readiness Index) and higher technology proficiency (as measured by the Mobile Device Proficiency Questionnaire) were associated with more positive technology attitudes toward the technologies to support health and cognition. Also, contrary to expectations, poorer health was associated with less positive attitudes toward the described technologies.

**Table 1:** Relations among variables.

	Positive attitudes	Privacy concerns	Mobile device proficiency	Technology readiness	Health literacy	Health	Cognitive deficits	Age	Gender
Positive attitudes	—								
Privacy concerns	0.000	—							
Mobile device proficiency	0.223*	−0.101	—						
Technology readiness	0.494 ***	−0.183	0.615***	—					
Health literacy	0.004	0.001	0.190	0.189	—				
Health	0.335**	−0.053	−0.035	−0.012	0.160	—			
Cognitive deficits	0.047	0.029	−0.239*	−0.253*	−0.255*	−0.141	—		
Age	−0.013	−0.007	−0.191	−0.201	−0.124	−0.080	0.105	—	
Gender	0.039	0.183	−0.196	−0.155	−0.329**	−0.006	0.179	0.144	—

*p < 0.05, **p < 0.01, ***p < 0.001. Significant relationships are depicted using bolding.

Table 2 presents Pearson correlations among the five attitudinal dimensions associated with each technology depicted in Figure 1. A few relationships are of note. Not surprisingly, a strong positive association was observed between participants’ interest in the described technologies and their willingness to adopt them. Perceived usefulness also correlated strongly with participants’ willingness to adopt. Perceptions of usefulness and interest also correlated with participants’ likelihood of recommending the technologies to others. Finally, negative associations (though weaker) were observed between privacy concerns and interest and willingness to adopt, as well as participants’ ratings of usefulness of the described technologies and whether participants would recommend these technologies to others.

**Table 2:** Relations among 5 primary outcomes.

	Useful	Recommend	Privacy concern	Interested	Adopt
Useful	—				
Recommend	0.895***	—			
Privacy concern	−0.316**	−0.290**	—		
Interested	0.654***	0.658***	−0.277**	—	
Adopt	0.766***	0.789***	−0.299**	0.904***	—

**p < 0.01, ***p < 0.001.

## Conclusion

Older adults adopt newer technologies at a slower rate compared to younger adults, and this can impact the adoption and use of healthcare technologies that might facilitate the detection of, and intervention for, age-related cognitive and health problems. However, the potential of these technologies for improving the health and independence of older adults depends crucially on adoption by older adults, which depends on willingness to adopt. This study examined older adults’ attitudes toward, and willingness to, adopt three different healthcare technologies to support health, cognition, and adherence to healthy behaviors. Remote monitoring technologies and their benefits can provide crucial solutions to the challenges of population aging and deliver efficient and effective healthcare during times of pandemic.

The main finding was that older adults, on average, had more positive than negative attitudes toward all three health-supporting technologies (however, this conclusion must be interpreted considering study limitations described later). This included agreeing that described technologies would be useful, that they would recommend the technologies to others, that they would be interested in learning more about each technology, and that they would consider adopting the technologies themselves. Positive attitudes contradict stereotypes that older adults are generally unwilling or afraid to use technology (Mitzner et al., 2010). When asked about whether data privacy should be a concern, participants’ responses were neutral, suggesting that they were not unconcerned about privacy issues, but also, not strongly concerned either. More nuanced and detailed questions about privacy might help unpack specific concerns and how these concerns impact adoption of these technologies.

Contrary to expectations, participants who read scenarios featuring individuals described as more vulnerable did not rate the described technologies as more useful, nor did they indicate less concern about data privacy. We anticipated that poorer health would enhance perceived usefulness of such technologies. Admittedly, this was a subtle manipulation, and it is possible that descriptions that made vulnerability more salient could have had an impact on participants’ ratings. Further, contrary to predictions, poorer health and cognition was not associated with more positive attitudes. In fact, poor overall health was associated with more negative attitudes toward these technologies. This finding is congruent with a recent, large-scale national survey in the United States finding that individuals with poorer health were less likely to use wearable health devices (Chandrasekaran et al., 2020). This finding is inconsistent, however, with the Senior Technology Acceptance Model (STAM) which predicts that individuals with poorer health are more likely to use technology to compensate for health limitations. However, associations may be different when considering health in relation to usage behaviors compared to attitudes, and general technology use compared to the specific technologies described in our hypothetical scenarios.

An important goal for researchers in this area is to develop interventions to reduce the age-related digital divide (Charness and Boot, 2022). Our study suggests a few important factors to target to help promote adoption. Positive attitudes toward adoption were predicted both by mobile device proficiency and general attitudes toward technology. These specific factors might be targeted through intervention to help promote adoption of health technologies like the ones described here. With respect to general attitudes toward technology, it is important to recognize that attitudes may not improve spontaneously over time (Lee et al., 2019). However, structural equation modeling of a large data set has suggested that enhancing technology proficiency can result in more positive attitudes, facilitating technology adoption and use (Czaja et al., 2006). Technology training interventions should be developed to target proficiency. Technology proficiency and familiarity likely impacts perceived ease of use, a critical factor related to intention to adopt new technologies according to several models of technology acceptance and adoption (e.g., Davis, 1989; Venkatesh et al., 2003; Chen and Chan, 2014). However, like technologies themselves, the design of instructional support and training programs to enhance proficiency should also consider the needs, preferences, and abilities of older adults (Czaja and Sharit, 2012; Cotten et al., 2016). It would be beneficial if this training could also account for individual differences in initial technology proficiency (Roque and Boot, 2018).

Privacy concerns were correlated with intention to adopt described technologies. This is consistent with the Extended Unified Theory of Acceptance and Use of Technology (UTAUT) model (Cimperman et al., 2016), which used questions like “I would feel totally safe providing sensitive personal information about myself over the Internet” to assess the construct of perceived security. Specifically, it was found that perceived security of data was an important predictor of behavioral intention to adopt telehealth solutions. Based on our findings, interventions might help address these concerns through education. Designers of these technologies should pay careful attention too to data privacy to help alleviate concerns.

These results, however, need to be interpreted considering several study limitations. The primary limitation was a sample that was mostly of high socioeconomic status (SES), and who answered surveys *via* an email (technology-based) invitation. This likely provided an overestimate of the broader population of older adults’ positive attitudes toward these technologies. Though, with 75% of older adults online in the United States at this point (Pew Research Center, 2022), our sample may not have been entirely unrepresentative of older adults in the United States with respect to technology experience. Also, older adults in our study were community dwelling, likely with few limitations of instrumental activities of daily living, and were generally of good physical and cognitive health. Because of a limited range of health and cognitive status scores, our results may have underestimated the importance of these variables in predicting attitudes among the broader population of older adults.

As with all survey studies, we had to balance the amount of information gathered from each participant (i.e., number of questions) with participant burden. First, the sample size was relatively small and did not allow for multivariate analyses. Further, we chose to prioritize concepts such as technology proficiency, general technology attitudes, health, and cognition. However, it should be acknowledged that other demographic variables (e.g., marital status, living context) and experiential variables (technology device ownership, previous device use) may also play critical roles in shaping adoption, use, and attitudes toward technology. Further, given the brief nature of the survey and scenarios described, we did not describe and evaluate participants’ responses to specific privacy and data security issues, or explore these issues in depth. Focus group studies are planned to provide a more nuanced and comprehensive review of these issues based on the results of this survey study. Finally, our data captured one snapshot in time; *change* in health status may be a particularly important factor to consider with respect to attitudes toward health technologies.

Despite these limitations, however, our results provide some initial insights into older adults’ attitudes toward novel health technologies and barriers to adoption. These insights have the potential to shape interventions to help ensure that useful technologies are widely adopted, providing benefits to the individual and to society. Future studies should examine these questions in larger, more diverse samples to provide additional insights, assess usage rather than behavioral intention, and focus groups have the potential to provide a more nuanced, qualitative understanding of barriers and facilitators related to adoption. For example, a planned focus group study of older adults with and without mild cognitive impairment (MCI) will present these same scenarios to participants to better understand perceived technology benefits, specific privacy concerns, and other attitudinal barriers and concerns not assessed in the current study, and whether these barriers and concerns might differ between participants who are more vulnerable (MCI participants) or less vulnerable to further health and cognitive decline. As part of a larger technology-based clinical trial examining adherence to home-based cognitive assessment, we also plan to have a subset of participants wear a smartwatch for multiple months to better understand factors related to not just intention, but actual usage.

## Data availability statement

The raw data supporting the conclusions of this article will be made available by the authors, without undue reservation.

## Ethics statement

The studies involving human participants were reviewed and approved by Florida State University Institutional Review Board. Written informed consent for participation was not required for this study in accordance with the national legislation and the institutional requirements.

## Author contributions

IF conceptualized this study, coded surveys into Qualtric, conducted data analyses, and completed the first draft of this manuscript. WB obtained ethical approval, assisted with data analysis, and revision of the manuscript. All authors contributed to the article and approved the submitted version.

## Funding

This work was supported through a National Institute on Aging Supplement to the Adherence Promotion with Person-centered Technology (APPT) Project—R01AG064529-04S1.

## Conflict of interest

The authors declare that the research was conducted in the absence of any commercial or financial relationships that could be construed as a potential conflict of interest.

## Publisher’s note

All claims expressed in this article are solely those of the authors and do not necessarily represent those of their affiliated organizations, or those of the publisher, the editors and the reviewers. Any product that may be evaluated in this article, or claim that may be made by its manufacturer, is not guaranteed or endorsed by the publisher.

## Footnotes

^1^ https://isl.fsu.edu/
