# Supplementary material for: Understanding older adults’ attitudes toward mobile and wearable technologies to support health and cognition
Source: Front Psychol. 2022 Dec 8;13:1036092. doi: 10.3389/fpsyg.2022.1036092 (PMC9779945; doi:10.3389/fpsyg.2022.1036092)
Supplement: Supplementary file 1 [file Presentation_1.pdf]

Supplementary Material

This section presents the wording of the three hypothetical health technology scenarios used in the current study. Each scenario had two versions, one in which basic information was provided about the individual who might be using the technology (name, gender, age), and a version in which additional information was provided to suggest the person was vulnerable to health challenges associated with the purpose of the technology. We also provide images of the technologies shown to participants, and the exact questions participants were asked about each technology.

Non-Vulnerable Scenarios

Case Scenario One - Assessing Cognitive Decline

Cindy is a 65-year-old woman. She recently learned of a digital watch that can be worn on the wrist that can collect data about her day-to-day activities. This information collected over a sufficiently long period can help generate predictions about whether she will experience serious cognitive problems in the future, for example, future memory problems.

Case Scenario Two - Supporting Health Behaviors

Jimmy is a 69-year-old man. He recently learned of a digital watch that can be worn on the wrist that can collect data about his day-to-day activities. This information collected over a sufficiently long period can help generate predictions about his daily routine and provide encouragement to engage in healthy activities, such as exercise, at a time when the technology predicts he has the free time to do so.

Case Scenario Three - Understanding and Predicting Health

Mindy is a 72-year-old woman. She recently received a newsletter from her healthcare provider encouraging her to download a new health app for her phone that would allow her to complete short health surveys every so often to help her and her physician understand her current health status and predict future health problems.

Vulnerable Scenarios

Case Scenario One - Assessing Cognitive Decline
Cindy is a 65-year-old woman with a family history of Alzheimer's disease. She recently learned of a digital watch that can be worn on the wrist that can collect data about her day-to-day activities. This information collected over a sufficiently long period can help generate predictions about whether she will experience serious cognitive problems in the future, for example, future memory problems.

Case Scenario Two - Supporting Health Behaviors

Jimmy is a 69-year-old man who struggles to maintain healthy habits. He recently learned of a digital watch that can be worn on the wrist that can collect data about his day-to-day activities. This information collected over a sufficiently long period can help generate predictions about his daily routine and provide encouragement to engage in healthy activities, such as exercise, at a time when the technology predicts he has the free time to do so.

Case Scenario Three - Understanding and Predicting Health

Mindy is a 72-year-old woman living with multiple chronic conditions (e.g., high blood pressure and diabetes). She recently received a newsletter from her healthcare provider encouraging her to download a new health app for her phone that would allow her to complete short health surveys every so often to help her and her physician understand her current health status and predict future health problems.

1.1 Supplementary Figure

[Image of smartwatch removed due to copyright. The figure is available from the authors upon request.]

Supplementary Figure 1 shows the image of a smartwatch that was shown to participants taking the survey as they responded to Scenarios 1 and 2.

1.2 Supplementary Figure

[Image of smartphone survey removed due to copyright. The figure is available from the authors upon request.]

Supplementary Figure 2 shows the image of a smartphone that was shown to participants as they responded to Scenario 3.

1.1 Supplementary Table

	Questions	Strongly Disagree	Disagree	Neutral	Agree	Strongly Agree
1	This piece of technology would be useful for [Name].					
2	I would recommend that [Name] adopt and use this technology.					
3	[Name] should be concerned about collecting and sharing this type of data.					
4	I am interested in learning more about this type of technology.					
5	I would consider adopting and using this technology.					

Supplementary Table 1.1 showing the questions that were used to evaluate each of the case scenarios.
